# Measurement of the ${B} _{c} ^+$ meson lifetime using ${B} _{c} ^+ \rightarrow {{J} /\psi } \mu ^+ \upnu _\mu X$ decays

**DOI:** 10.1140/epjc/s10052-014-2839-x

**Published:** 2014-05-01

**Authors:** The LHCb Collaboration, R. Aaij, B. Adeva, M. Adinolfi, A. Affolder, Z. Ajaltouni, J. Albrecht, F. Alessio, M. Alexander, S. Ali, G. Alkhazov, P. Alvarez Cartelle, A. A. Alves, S. Amato, S. Amerio, Y. Amhis, L. Anderlini, J. Anderson, R. Andreassen, M. Andreotti, J. E. Andrews, R. B. Appleby, O. Aquines Gutierrez, F. Archilli, A. Artamonov, M. Artuso, E. Aslanides, G. Auriemma, M. Baalouch, S. Bachmann, J. J. Back, A. Badalov, V. Balagura, W. Baldini, R. J. Barlow, C. Barschel, S. Barsuk, W. Barter, V. Batozskaya, Th. Bauer, A. Bay, J. Beddow, F. Bedeschi, I. Bediaga, S. Belogurov, K. Belous, I. Belyaev, E. Ben-Haim, G. Bencivenni, S. Benson, J. Benton, A. Berezhnoy, R. Bernet, M.-O. Bettler, M. van Beuzekom, A. Bien, S. Bifani, T. Bird, A. Bizzeti, P. M. Bjørnstad, T. Blake, F. Blanc, J. Blouw, S. Blusk, V. Bocci, A. Bondar, N. Bondar, W. Bonivento, S. Borghi, A. Borgia, M. Borsato, T. J. V. Bowcock, E. Bowen, C. Bozzi, T. Brambach, J. van den Brand, J. Bressieux, D. Brett, M. Britsch, T. Britton, N. H. Brook, H. Brown, A. Bursche, G. Busetto, J. Buytaert, S. Cadeddu, R. Calabrese, O. Callot, M. Calvi, M. Calvo Gomez, A. Camboni, P. Campana, D. Campora Perez, A. Carbone, G. Carboni, R. Cardinale, A. Cardini, H. Carranza-Mejia, L. Carson, K. Carvalho Akiba, G. Casse, L. Castillo Garcia, M. Cattaneo, Ch. Cauet, R. Cenci, M. Charles, Ph. Charpentier, S.-F. Cheung, N. Chiapolini, M. Chrzaszcz, K. Ciba, X. Cid Vidal, G. Ciezarek, P. E. L. Clarke, M. Clemencic, H. V. Cliff, J. Closier, C. Coca, V. Coco, J. Cogan, E. Cogneras, P. Collins, A. Comerma-Montells, A. Contu, A. Cook, M. Coombes, S. Coquereau, G. Corti, I. Counts, B. Couturier, G. A. Cowan, D. C. Craik, M. Cruz Torres, S. Cunliffe, R. Currie, C. D’Ambrosio, J. Dalseno, P. David, P. N. Y. David, A. Davis, I. De Bonis, K. De Bruyn, S. De Capua, M. De Cian, J. M. De Miranda, L. De Paula, W. De Silva, P. De Simone, D. Decamp, M. Deckenhoff, L. Del Buono, N. Déléage, D. Derkach, O. Deschamps, F. Dettori, A. Di Canto, H. Dijkstra, S. Donleavy, F. Dordei, M. Dorigo, P. Dorosz, A. Dosil Suárez, D. Dossett, A. Dovbnya, F. Dupertuis, P. Durante, R. Dzhelyadin, A. Dziurda, A. Dzyuba, S. Easo, U. Egede, V. Egorychev, S. Eidelman, S. Eisenhardt, U. Eitschberger, R. Ekelhof, L. Eklund, I. El Rifai, Ch. Elsasser, A. Falabella, C. Färber, C. Farinelli, S. Farry, D. Ferguson, V. Fernandez Albor, F. Ferreira Rodrigues, M. Ferro-Luzzi, S. Filippov, M. Fiore, M. Fiorini, C. Fitzpatrick, M. Fontana, F. Fontanelli, R. Forty, O. Francisco, M. Frank, C. Frei, M. Frosini, E. Furfaro, A. Gallas Torreira, D. Galli, M. Gandelman, P. Gandini, Y. Gao, J. Garofoli, J. Garra Tico, L. Garrido, C. Gaspar, R. Gauld, E. Gersabeck, M. Gersabeck, T. Gershon, Ph. Ghez, A. Gianelle, V. Gibson, L. Giubega, V. V. Gligorov, C. Göbel, D. Golubkov, A. Golutvin, A. Gomes, H. Gordon, M. Grabalosa Gándara, R. Graciani Diaz, L. A. Granado Cardoso, E. Graugés, G. Graziani, A. Grecu, E. Greening, S. Gregson, P. Griffith, L. Grillo, O. Grünberg, B. Gui, E. Gushchin, Yu. Guz, T. Gys, C. Hadjivasiliou, G. Haefeli, C. Haen, T. W. Hafkenscheid, S. C. Haines, S. Hall, B. Hamilton, T. Hampson, S. Hansmann-Menzemer, N. Harnew, S. T. Harnew, J. Harrison, T. Hartmann, J. He, T. Head, V. Heijne, K. Hennessy, P. Henrard, J. A. Hernando Morata, E. van Herwijnen, M. Heß, A. Hicheur, D. Hill, M. Hoballah, C. Hombach, W. Hulsbergen, P. Hunt, T. Huse, N. Hussain, D. Hutchcroft, D. Hynds, V. Iakovenko, M. Idzik, P. Ilten, R. Jacobsson, A. Jaeger, E. Jans, P. Jaton, A. Jawahery, F. Jing, M. John, D. Johnson, C. R. Jones, C. Joram, B. Jost, N. Jurik, M. Kaballo, S. Kandybei, W. Kanso, M. Karacson, T. M. Karbach, I. R. Kenyon, T. Ketel, B. Khanji, C. Khurewathanakul, S. Klaver, O. Kochebina, I. Komarov, R. F. Koopman, P. Koppenburg, M. Korolev, A. Kozlinskiy, L. Kravchuk, K. Kreplin, M. Kreps, G. Krocker, P. Krokovny, F. Kruse, M. Kucharczyk, V. Kudryavtsev, K. Kurek, T. Kvaratskheliya, V. N. La Thi, D. Lacarrere, G. Lafferty, A. Lai, D. Lambert, R. W. Lambert, E. Lanciotti, G. Lanfranchi, C. Langenbruch, T. Latham, C. Lazzeroni, R. Le Gac, J. van Leerdam, J.-P. Lees, R. Lefèvre, A. Leflat, J. Lefrançois, S. Leo, O. Leroy, T. Lesiak, B. Leverington, Y. Li, M. Liles, R. Lindner, C. Linn, F. Lionetto, B. Liu, G. Liu, S. Lohn, I. Longstaff, J. H. Lopes, N. Lopez-March, P. Lowdon, H. Lu, D. Lucchesi, J. Luisier, H. Luo, E. Luppi, O. Lupton, F. Machefert, I. V. Machikhiliyan, F. Maciuc, O. Maev, S. Malde, G. Manca, G. Mancinelli, M. Manzali, J. Maratas, U. Marconi, P. Marino, R. Märki, J. Marks, G. Martellotti, A. Martens, A. Martín Sánchez, M. Martinelli, D. Martinez Santos, D. Martins Tostes, A. Massafferri, R. Matev, Z. Mathe, C. Matteuzzi, A. Mazurov, M. McCann, J. McCarthy, A. McNab, R. McNulty, B. McSkelly, B. Meadows, F. Meier, M. Meissner, M. Merk, D. A. Milanes, M.-N. Minard, J. Molina Rodriguez, S. Monteil, D. Moran, M. Morandin, P. Morawski, A. Mordà, M. J. Morello, R. Mountain, I. Mous, F. Muheim, K. Müller, R. Muresan, B. Muryn, B. Muster, P. Naik, T. Nakada, R. Nandakumar, I. Nasteva, M. Needham, S. Neubert, N. Neufeld, A. D. Nguyen, T. D. Nguyen, C. Nguyen-Mau, M. Nicol, V. Niess, R. Niet, N. Nikitin, T. Nikodem, A. Novoselov, A. Oblakowska-Mucha, V. Obraztsov, S. Oggero, S. Ogilvy, O. Okhrimenko, R. Oldeman, G. Onderwater, M. Orlandea, J. M. Otalora Goicochea, P. Owen, A. Oyanguren, B. K. Pal, A. Palano, M. Palutan, J. Panman, A. Papanestis, M. Pappagallo, L. Pappalardo, C. Parkes, C. J. Parkinson, G. Passaleva, G. D. Patel, M. Patel, C. Patrignani, C. Pavel-Nicorescu, A. Pazos Alvarez, A. Pearce, A. Pellegrino, G. Penso, M. Pepe Altarelli, S. Perazzini, E. Perez Trigo, P. Perret, M. Perrin-Terrin, L. Pescatore, E. Pesen, G. Pessina, K. Petridis, A. Petrolini, E. Picatoste Olloqui, B. Pietrzyk, T. Pilař, D. Pinci, A. Pistone, S. Playfer, M. Plo Casasus, F. Polci, G. Polok, A. Poluektov, E. Polycarpo, A. Popov, D. Popov, B. Popovici, C. Potterat, A. Powell, J. Prisciandaro, A. Pritchard, C. Prouve, V. Pugatch, A. Puig Navarro, G. Punzi, W. Qian, B. Rachwal, J. H. Rademacker, B. Rakotomiaramanana, M. Rama, M. S. Rangel, I. Raniuk, N. Rauschmayr, G. Raven, S. Redford, S. Reichert, M. M. Reid, A. C. dos Reis, S. Ricciardi, A. Richards, K. Rinnert, V. Rives Molina, D. A. Roa Romero, P. Robbe, D. A. Roberts, A. B. Rodrigues, E. Rodrigues, P. Rodriguez Perez, S. Roiser, V. Romanovsky, A. Romero Vidal, M. Rotondo, J. Rouvinet, T. Ruf, F. Ruffini, H. Ruiz, P. Ruiz Valls, G. Sabatino, J. J. Saborido Silva, N. Sagidova, P. Sail, B. Saitta, V. Salustino Guimaraes, B. Sanmartin Sedes, R. Santacesaria, C. Santamarina Rios, E. Santovetti, M. Sapunov, A. Sarti, C. Satriano, A. Satta, M. Savrie, D. Savrina, M. Schiller, H. Schindler, M. Schlupp, M. Schmelling, B. Schmidt, O. Schneider, A. Schopper, M.-H. Schune, R. Schwemmer, B. Sciascia, A. Sciubba, M. Seco, A. Semennikov, K. Senderowska, I. Sepp, N. Serra, J. Serrano, P. Seyfert, M. Shapkin, I. Shapoval, Y. Shcheglov, T. Shears, L. Shekhtman, O. Shevchenko, V. Shevchenko, A. Shires, R. Silva Coutinho, G. Simi, M. Sirendi, N. Skidmore, T. Skwarnicki, N. A. Smith, E. Smith, E. Smith, J. Smith, M. Smith, H. Snoek, M. D. Sokoloff, F. J. P. Soler, F. Soomro, D. Souza, B. Souza De Paula, B. Spaan, A. Sparkes, F. Spinella, P. Spradlin, F. Stagni, S. Stahl, O. Steinkamp, S. Stevenson, S. Stoica, S. Stone, B. Storaci, S. Stracka, M. Straticiuc, U. Straumann, R. Stroili, V. K. Subbiah, L. Sun, W. Sutcliffe, S. Swientek, V. Syropoulos, M. Szczekowski, P. Szczypka, D. Szilard, T. Szumlak, S. T’Jampens, M. Teklishyn, G. Tellarini, E. Teodorescu, F. Teubert, C. Thomas, E. Thomas, J. van Tilburg, V. Tisserand, M. Tobin, S. Tolk, L. Tomassetti, D. Tonelli, S. Topp-Joergensen, N. Torr, E. Tournefier, S. Tourneur, M. T. Tran, M. Tresch, A. Tsaregorodtsev, P. Tsopelas, N. Tuning, M. Ubeda Garcia, A. Ukleja, A. Ustyuzhanin, U. Uwer, V. Vagnoni, G. Valenti, A. Vallier, R. Vazquez Gomez, P. Vazquez Regueiro, C. Vázquez Sierra, S. Vecchi, J. J. Velthuis, M. Veltri, G. Veneziano, M. Vesterinen, B. Viaud, D. Vieira, X. Vilasis-Cardona, A. Vollhardt, D. Volyanskyy, D. Voong, A. Vorobyev, V. Vorobyev, C. Voß, H. Voss, J. A. de Vries, R. Waldi, C. Wallace, R. Wallace, S. Wandernoth, J. Wang, D. R. Ward, N. K. Watson, A. D. Webber, D. Websdale, M. Whitehead, J. Wicht, J. Wiechczynski, D. Wiedner, L. Wiggers, G. Wilkinson, M. P. Williams, M. Williams, F. F. Wilson, J. Wimberley, J. Wishahi, W. Wislicki, M. Witek, G. Wormser, S. A. Wotton, S. Wright, S. Wu, K. Wyllie, Y. Xie, Z. Xing, Z. Yang, X. Yuan, O. Yushchenko, M. Zangoli, M. Zavertyaev, F. Zhang, L. Zhang, W. C. Zhang, Y. Zhang, A. Zhelezov, A. Zhokhov, L. Zhong, A. Zvyagin

**Affiliations:** 1 Centro Brasileiro de Pesquisas Físicas (CBPF), Rio de Janeiro, Brazil; 2 Universidade Federal do Rio de Janeiro (UFRJ), Rio de Janeiro, Brazil; 3 Center for High Energy Physics, Tsinghua University, Beijing, China; 4 LAPP, Université de Savoie, CNRS/IN2P3, Annecy-Le-Vieux, France; 5 Clermont Université, Université Blaise Pascal, CNRS/IN2P3, LPC, Clermont-Ferrand, France; 6 CPPM, Aix-Marseille Université, CNRS/IN2P3, Marseille, France; 7 LAL, Université Paris-Sud, CNRS/IN2P3, Orsay, France; 8 LPNHE, Université Pierre et Marie Curie, Université Paris Diderot, CNRS/IN2P3, Paris, France; 9 Fakultät Physik, Technische Universität Dortmund, Dortmund, Germany; 10 Max-Planck-Institut für Kernphysik (MPIK), Heidelberg, Germany; 11 Physikalisches Institut, Ruprecht-Karls-Universität Heidelberg, Heidelberg, Germany; 12 School of Physics, University College Dublin, Dublin, Ireland; 13 Sezione INFN di Bari, Bari, Italy; 14 Sezione INFN di Bologna, Bologna, Italy; 15 Sezione INFN di Cagliari, Cagliari, Italy; 16 Sezione INFN di Ferrara, Ferrara, Italy; 17 Sezione INFN di Firenze, Florence, Italy; 18 Laboratori Nazionali dell’INFN di Frascati, Frascati, Italy; 19 Sezione INFN di Genova, Genoa, Italy; 20 Sezione INFN di Milano Bicocca, Milan, Italy; 21 Sezione INFN di Padova, Padua, Italy; 22 Sezione INFN di Pisa, Pisa, Italy; 23 Sezione INFN di Roma Tor Vergata, Rome, Italy; 24 Sezione INFN di Roma La Sapienza, Rome, Italy; 25 Henryk Niewodniczanski Institute of Nuclear Physics Polish Academy of Sciences, Kraków, Poland; 26 Faculty of Physics and Applied Computer Science, AGH, University of Science and Technology, Kraków, Poland; 27 National Center for Nuclear Research (NCBJ), Warsaw, Poland; 28 Horia Hulubei National Institute of Physics and Nuclear Engineering, Bucharest-Magurele, Romania; 29 Petersburg Nuclear Physics Institute (PNPI), Gatchina, Russia; 30 Institute of Theoretical and Experimental Physics (ITEP), Moscow, Russia; 31 Institute of Nuclear Physics, Moscow State University (SINP MSU), Moscow, Russia; 32 Institute for Nuclear Research of the Russian Academy of Sciences (INR RAN), Moscow, Russia; 33 Budker Institute of Nuclear Physics (SB RAS) and Novosibirsk State University, Novosibirsk, Russia; 34 Institute for High Energy Physics (IHEP), Protvino, Russia; 35 Universitat de Barcelona, Barcelona, Spain; 36 Universidad de Santiago de Compostela, Santiago de Compostela, Spain; 37 European Organization for Nuclear Research (CERN), Geneva, Switzerland; 38 Ecole Polytechnique Fédérale de Lausanne (EPFL), Lausanne, Switzerland; 39 Physik-Institut, Universität Zürich, Zurich, Switzerland; 40 Nikhef National Institute for Subatomic Physics, Amsterdam, The Netherlands; 41 Nikhef National Institute for Subatomic Physics and VU University Amsterdam, Amsterdam, The Netherlands; 42 NSC Kharkiv Institute of Physics and Technology (NSC KIPT), Kharkiv, Ukraine; 43 Institute for Nuclear Research of the National Academy of Sciences (KINR), Kiev, Ukraine; 44 University of Birmingham, Birmingham, UK; 45 H.H. Wills Physics Laboratory, University of Bristol, Bristol, UK; 46 Cavendish Laboratory, University of Cambridge, Cambridge, UK; 47 Department of Physics, University of Warwick, Coventry, UK; 48 STFC Rutherford Appleton Laboratory, Didcot, UK; 49 School of Physics and Astronomy, University of Edinburgh, Edinburgh, United Kingdom; 50 School of Physics and Astronomy, University of Glasgow, Glasgow, UK; 51 Oliver Lodge Laboratory, University of Liverpool, Liverpool, UK; 52 Imperial College London, London, UK; 53 School of Physics and Astronomy, University of Manchester, Manchester, UK; 54 Department of Physics, University of Oxford, Oxford, UK; 55 Massachusetts Institute of Technology, Cambridge, MA USA; 56 University of Cincinnati, Cincinnati, OH USA; 57 University of Maryland, College Park, MD USA; 58 Syracuse University, Syracuse, NY USA; 59 Pontifícia Universidade Católica do Rio de Janeiro (PUC-Rio), Rio de Janeiro, Brazil; 60 Institut für Physik, Universität Rostock, Rostock, Germany; 61 National Research Centre Kurchatov Institute, Moscow, Russia; 62 KVI, University of Groningen, Groningen, The Netherlands; 63 Celal Bayar University, Manisa, Turkey; 64 CERN, 1211 Geneva 23, Switzerland

## Abstract

The lifetime of the ${B} _{c} ^+$ meson is measured using semileptonic decays having a $J/\psi$ meson and a muon in the final state. The data, corresponding to an integrated luminosity of $2 \text{ fb }^{-1}$, are collected by the LHCb detector in $pp$ collisions at a centre-of-mass energy of 8 TeV. The measured lifetime is

$$\begin{aligned} \tau = 509\pm 8\pm 12\mathrm {~fs}, \end{aligned}$$

where the first uncertainty is statistical and the second is systematic.

## Introduction

The $${B} _{c} ^+$$ meson, formed of a $$\overline{{b} }$$ and a $${c} $$ quark, is an excellent laboratory to study QCD and weak interactions.^1^ The $${B} _{c} ^+$$ meson bound-state dynamics can be treated in a non-relativistic expansion by QCD-inspired effective models that successfully describe the spectroscopy of quarkonia. However, $${B} _{c} ^+$$ production and decay dynamics have some distinctive features, since this meson is the only observed open-flavour state formed by two heavy quarks. The decay proceeds through the weak interaction, and about 70% of the width is expected to be due to the CKM favoured $$c \rightarrow s$$ transition [1]. This decay process, challenging to detect, has recently been observed in the $${B} _{c} ^+ \rightarrow {B} ^0_{s} \pi ^+ $$ mode by the LHCb collaboration [2]. The $$b \rightarrow c$$ transition offers an easier experimental signature, having a substantial probability to produce a $${{J} /\psi }$$ meson. Indeed, the $${B} _{c} ^+$$ meson was discovered by the CDF collaboration [3] through the observation of the $${B} _{c} ^+ \rightarrow {{J} /\psi } \ell ^+\nu _{\ell }X ~(\ell = \mu , e)$$ semileptonic decays, where $$X$$ denotes any possible additional particles in the final state.

The precise measurement of the ${B} _{c} ^+$ lifetime provides an essential test of the theoretical models describing its dynamics. Computations based on various frameworks [1, 4–9] predict values ranging from 300 to 700 fs. The world average value of the ${B} _{c} ^+$ lifetime reported by the PDG in 2013 [10] is 452 $\pm$ 33 fs. This was obtained from measurements performed at the Tevatron, using semileptonic decays [3, 11, 12] and the rarer $B_c^+ \rightarrow {{J} /\psi } \pi ^+$ decay [13].

The unprecedented ${B} _{c} ^+$ production rate achieved at the LHC has thus far been used to measure many ${B} _{c} ^+$ decay properties, with several new decay modes observed by LHCb   [2, 14–18]. The current knowledge of the lifetime is one of the largest systematic uncertainties in the relative branching fraction measurements, also affecting the determination of the production cross-section [19]. This paper reports a measurement of the ${B} _{c} ^+$ lifetime using the semileptonic decays ${B} _{c} ^+ \rightarrow {{J} /\psi } \mu ^+ \upnu _\mu X$ with ${{J} /\psi } \rightarrow \mu ^+\mu ^-$.

## Detector and data sample

The LHCb detector [20] is a single-arm forward spectrometer covering the pseudorapidity range $$2<\eta <5$$, designed for the study of particles containing $${b} $$ or $${c} $$ quarks. The detector includes a high-precision tracking system consisting of a silicon-strip vertex detector surrounding the $$pp$$ interaction region, a large-area silicon-strip detector located upstream of a dipole magnet with a bending power of about $$4\mathrm {\,T\cdot m}$$, and three stations of silicon-strip detectors and straw drift tubes placed downstream. The combined tracking system provides a momentum measurement with relative uncertainty that varies from 0.4% at 5 $${\mathrm {GeV\!/}c}$$ to 0.6% at 100 $${\mathrm {GeV\!/}c}$$, and impact parameter resolution of 20$${\,\upmu \mathrm m}$$ for tracks with large transverse momentum. Different types of charged hadrons are distinguished by information from two ring-imaging Cherenkov detectors [21]. Photon, electron and hadron candidates are identified by a calorimeter system consisting of scintillating-pad and preshower detectors, an electromagnetic calorimeter and a hadronic calorimeter. Muons are identified by a system composed of alternating layers of iron and multiwire proportional chambers [22]. The trigger [23] consists of a hardware stage, based on information from the calorimeter and muon systems, followed by a software stage, which applies a full event reconstruction.

The analysis is performed on a data sample of $pp$ collisions at a centre-of-mass energy of 8 TeV, collected during 2012 and corresponding to an integrated luminosity of 2 fb$^{-1}$. Simulated event samples are generated for the signal decays and the decay modes contributing to the background. In the simulation, $pp$ collisions are generated using Pythia  [24] with a specific LHCb configuration [25]. The production of ${B} _{c} ^+$ mesons, which is not adequately simulated in Pythia, is performed by the dedicated generator Bcvegpy [26] using a ${B} _{c} ^+$ mass of 6276 ${\mathrm {\,MeV\!/}c^2}$ and a lifetime of 450 fs. Several dynamical models are used to simulate ${B} _{c} ^+ \rightarrow {{J} /\psi } \mu ^+ \upnu _\mu$ decays, as discussed in Sect. 4. Decays of hadronic particles are described by EvtGen  [27], in which final state radiation is generated using Photos  [28]. The interaction of the generated particles with the detector and its response are implemented using the Geant4 toolkit [29, 30] as described in Ref. [31].

## Analysis strategy and event selection

Candidate signal decays are obtained from combinations of a dimuon compatible with a ${{J} /\psi }$ decay and an additional candidate muon track, denoted as a *bachelor* muon in the following, originating from a common vertex.

Since the expected signal yield is about $$10^4$$ candidates over a moderate background, the event selection and analysis are driven by the need to minimise systematic uncertainties. Selection variables that bias the $${B} _{c} ^+$$ candidate decay time distribution are avoided, and the selection is designed not only to suppress the background contributions, but also to allow their modelling using data. Background candidates with decay time and $${{J} /\psi } \mu $$ mass values comparable to the signal decays are mainly expected from $${b} $$-hadron decays to a $${{J} /\psi }$$ meson and a hadron that is misidentified as a muon. This misidentification background is modelled using data in which $${B} _{c} ^+$$ candidates are selected without any bias related to the identification of the bachelor muon. The candidate events are required to pass a trigger decision based solely on the information from the $${{J} /\psi }$$ $$\rightarrow $$ $$\mu ^+\mu ^-$$ candidate. To pass the hardware trigger, one or both tracks from the $${{J} /\psi }$$ decay must be identified as muons. In the first case, the muon is required to have a transverse momentum, $$p_{\mathrm{T}} $$, greater than $$1.48~{\mathrm {GeV\!/}c} $$, while in the second case, the product of the two $$p_{\mathrm{T}} $$ values must be larger than $$1.68~\mathrm {\,GeV} ^2/c^2$$. The software trigger selects dimuon candidates consistent with the decay of a $${{J} /\psi }$$ meson by applying loose criteria on the dimuon mass, vertex quality and muon identification, and requires $$p_{\mathrm{T}} > 2~{\mathrm {GeV\!/}c} $$.

An offline selection applies further kinematic criteria to enhance the signal purity. Requirements on the minimum $p_{\mathrm{T}}$ are applied to the two ${{J} /\psi }$ decay products ($1.4~{\mathrm {GeV\!/}c}$), the ${{J} /\psi }$ candidate ($2~{\mathrm {GeV\!/}c}$), the bachelor muon ($2.5~{\mathrm {GeV\!/}c}$) and the ${{J} /\psi } \mu$ combination ($6~{\mathrm {GeV\!/}c}$). The momentum of the bachelor muon must be between 13 and 150 ${\mathrm {GeV\!/}c}$. The ${{J} /\psi }$ candidate mass is required to be between 3.066 and 3.131 ${\mathrm {\,GeV\!/}c^2}$, a range corresponding to about four times the mass resolution. Two sideband mass regions, 3.005–3.036 and 3.156–3.190 ${\mathrm {\,GeV\!/}c^2}$, are used to evaluate the background from track pairs misidentified as ${{J} /\psi }$ candidates. The three muons are required to originate from a common vertex, with a $\chi ^2$ per degree of freedom from the fit smaller than 3.0. This restrictive requirement suppresses combinatorial background from random associations of real ${{J} /\psi }$ and muon candidates not originating from the same vertex. The ${{J} /\psi } \mu$ mass, $M_{{{J} /\psi } \mu }$, is reconstructed from a kinematic fit constraining the ${{J} /\psi }$ mass to its known value [10], and is required to be between 3.5 and 6.25 ${\mathrm {\,GeV\!/}c^2}$.

Particle identification is based on the information from the Cherenkov, calorimeter and muon detectors, combined into likelihood functions. The selection is based on the logarithm of the likelihood ratio, $\mathrm {DLL} _{P/P^{'}}$, for two given charged-particle hypotheses $P$ and $P^{'}$ among $\mu , \pi , K$ and $p$. The requirement $\mathrm {DLL} _{\mu /\pi }>1$ is applied on the two muon tracks forming the ${{J} /\psi }$ candidate. Dedicated, more restrictive identification requirements are applied to the bachelor muon candidate, including the criterion that the track is matched with muon detector hits in all stations downstream of the calorimeters. A track fit based on a Kalman filter [32] is performed using such hits, and the resulting $\chi ^2$ per degree of freedom must be lower than 1.5. Vetoes against the pion ($\mathrm {DLL} _{\mu /\pi }>3$), kaon ($\mathrm {DLL} _{K/\pi }<8$) and proton ($\mathrm {DLL} _{p/\pi }<20$) hypotheses are also applied. To avoid cases in which two candidate tracks are reconstructed from the same muon, the bachelor candidate is required not to share any hits in the muon detectors, and share less than 20% of hits in the tracking stations, with either of the two other muon candidates in the decay. Studies using simulated samples indicate that, after these requirements, the misidentified candidates are dominated by kaons and pions decaying in flight. Decays occurring in the tracker region are reduced by requiring a good match between the track segments reconstructed upstream and downstream of the magnet ($\chi ^2 <15.0$ with five degrees of freedom). The total selection efficiency for signal events, including the detector geometrical acceptance and the trigger, reconstruction and offline selection efficiencies, is predicted from simulation to be about 0.25%.

The selected sample consists of 29 756 candidates. Among the selected events, 0.6% have multiple candidates which, in most cases, are formed by the same three tracks where the muons with the same charge are exchanged. All candidates are retained, and this effect is considered as a potential source of systematic uncertainty.

To study the decay time distribution, a pseudo-proper time is determined for each candidate, defined as

1 $$\begin{aligned} t_{\mathrm {ps}} = \varvec{p} \cdot (\varvec{v}-\varvec{x}) \frac{M_{3\mu }}{|\varvec{p}|^2}, \end{aligned}$$

where $\varvec{p}$ is the three-momentum of the ${{J} /\psi } \mu$ system in the laboratory frame, and $\varvec{v}$ and $\varvec{x}$ are the measured positions of the ${B} _{c} ^+$ decay and production vertices, respectively. The primary $pp$ interaction vertex (PV) associated with the production of each ${B} _{c} ^+$ candidate is chosen as the one yielding the smallest difference in $\chi ^2$ when fitted with and without the ${B} _{c} ^+$ candidate. The position obtained from the latter fit is used. The three-$\mu$ mass $M_{3\mu }$ used in Eq. 1 is computed without the constraint on the ${{J} /\psi }$ mass, to reduce the potential bias from momentum scale miscalibration, which approximately cancels in the $M_{3\mu }/|\varvec{p}|$ ratio.

The ${B} _{c} ^+$ lifetime is determined using the variables $t_{\mathrm {ps}}$ and $M_{{{J} /\psi } \mu }$. To infer the ${B} _{c} ^+$ decay time from the pseudo-proper time , a statistical correction based on simulation, commonly referred to as the $k$-factor method, is adopted. There, the average effect of the momentum of the unreconstructed decay products on the determination of the ${B} _{c} ^+$ decay time is computed as a function of $M_{{{J} /\psi } \mu }$. The ${B} _{c} ^+$ momentum can also be reconstructed for each decay, up to a two-fold ambiguity, using the measured flight direction of the ${B} _{c} ^+$ meson and the knowledge of its mass. However, due to the short ${B} _{c} ^+$ lifetime, the achievable resolution is poor and strongly dependent on the decay time. Therefore, this partial reconstruction is not used in the lifetime determination to avoid potentially large biases, but exploited to study systematic uncertainties arising from the assumed kinematic model of the signal.

The background contributions are also modelled in the ($$t_{\mathrm {ps}}$$, $$M_{{{J} /\psi } \mu }$$) plane. Models are obtained from data whenever possible, with the notable exception of combinatorial background, whose contribution is inferred from large simulated samples of inclusive $${b} $$-hadron decays containing a $${{J} /\psi }$$ meson in the final state.

## Signal model

The expected ($t_{\mathrm {ps}}$, $M_{{{J} /\psi } \mu }$) distribution for the signal decays depends on the simulation of the dynamics for the ${B} _{c} ^+ \rightarrow {{J} /\psi } \mu ^+ \upnu _\mu$ decay and of the contributions from decay modes with additional particles in the final state (*feed-down* modes).

**Fig. 1:**
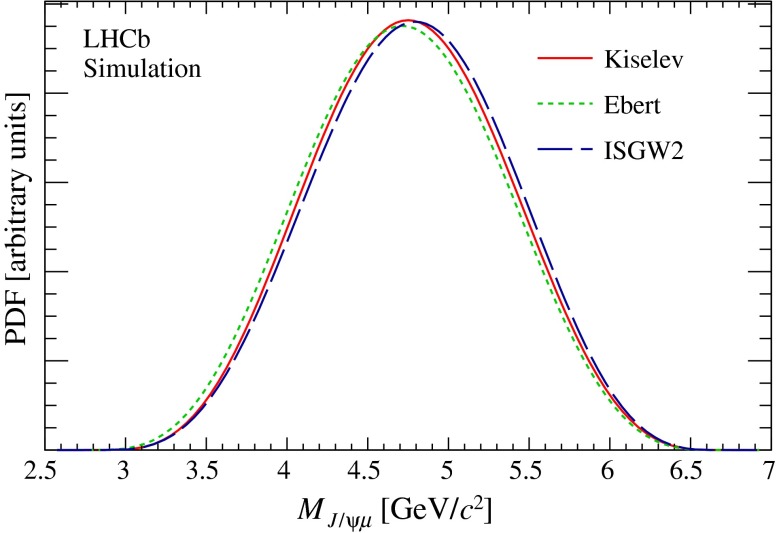
Distributions of $M_{{{J} /\psi } \mu }$, without simulation of detector response, for the Kiselev (*red solid line*), Ebert (*green short-dashed line*), and ISGW2 (*black long-dashed line*) models

For the ${B} _{c} ^+ \rightarrow {{J} /\psi } \mu ^+ \upnu _\mu$ decay, three different decay models are implemented in the simulation, referred to as Kiselev [33–35], Ebert [36] and ISGW2 [37]. The Kiselev model is adopted as the baseline and used to simulate more than 20 million events with the three muons in the nominal detector acceptance. Smaller samples generated with the alternative models are used for systematic studies. Figure 1 compares the probability density function (PDF) for $M_{{{J} /\psi } \mu }$ predicted by the three models, which exhibit only small differences with each other.

The simulation is used to predict the average ratio between the measured pseudo-proper time and the simulated true ${B} _{c} ^+$ decay time $t^*$. This correction term can be factorised as

2 $$\begin{aligned} k' \equiv \frac{t_{\mathrm {ps}}}{t^*} = \frac{t_{\mathrm {ps}}}{t_{\mathrm {ps}} ^*} \times \frac{t_{\mathrm {ps}} ^*}{t^*} \equiv \frac{t_{\mathrm {ps}}}{t_{\mathrm {ps}} ^*} \times k, \end{aligned}$$

where $t_{\mathrm {ps}} ^*$ is the simulated true value of the pseudo-proper time defined in Eq. 1. The $t_{\mathrm {ps}}/t_{\mathrm {ps}} ^*$ term accounts for imperfections in the experimental reconstruction, while the $k\equiv t_{\mathrm {ps}} ^*/t^*$ factor includes only the kinematic effects from unobserved particles in the final state. It is found that the kinematic term dominates the average deviation from unity and the r.m.s. width of the $k'$ variable. The $k$-factor distribution is empirically modelled from simulated events in bins of $M_{{{J} /\psi } \mu }$.

The resolution function describing $\Delta t\equiv t_{\mathrm {ps}}-t_{\mathrm {ps}} ^*$ is parametrised as the sum of three Gaussian functions with a common mean $t_0$ and different widths

3 $$\begin{aligned} G(\Delta t) = \sum _{i=1}^3 g_i ~\frac{1}{\sigma _i \sqrt{2\pi }} \exp \left( -\frac{(\Delta t-t_0)^2}{2\sigma _i^2}\right) . \end{aligned}$$

The parameters $g_i$, $t_0$ and $\sigma _i$ are determined from fits to the simulated events. A small bias $t_0=-1.9 \pm 0.2$ fs is found, and the core Gaussian term has parameters $g_1=0.74$, $\sigma _1=27$ fs. The other two Gaussian functions have parameters $g_2=0.24$, $\sigma _2=54$ fs, $g_3=0.02$, and $\sigma _3=260$ fs. These parameters are assumed not to depend on the decay time itself as indicated by the simulation. A fourth Gaussian term with the same mean and large width is added when performing the fit to simulated data to describe the small fraction of events having an incorrectly associated primary vertex. This is not included in the signal model because these events are considered as a background source, which is constrained from data by exploiting the negative tail of the $t_{\mathrm {ps}}$ distribution.

The model for the PDF of $t_{\mathrm {ps}} =kt^* + \Delta t$ is obtained for each $M_{{{J} /\psi } \mu }$ bin $m$ by convoluting the exponential $t^*$ distribution with the $k$-factor distribution $h^{m}(k)$ and the resolution function, resulting in

4 $$\begin{aligned}&f^m(t_{\mathrm {ps}}) \!=\! \sum _{i=1}^3 g_i \int \limits _{-\infty }^{+\infty }\mathrm dk\, h^{m}(k) \frac{1}{2k\tau }\nonumber \\&\quad \times \exp \left( \frac{\sigma _i^2}{2k^2 \tau ^2} \!-\! \frac{(t_{\mathrm {ps}} \! -\! t_{0} )}{k\tau }\right) \mathrm {erfc}\left( \frac{\sigma _i}{k\tau \sqrt{2}} \!-\! \frac{(t_{\mathrm {ps}} \!-\! t_{0} )}{\sigma _i\sqrt{2}}\right) ,\nonumber \\ \end{aligned}$$

where $\tau$ is the ${B} _{c} ^+$ lifetime and erfc is the complementary error function.

**Fig. 2:**
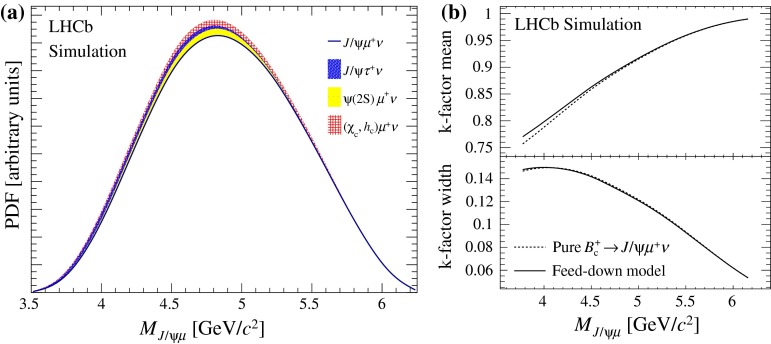
Corrections to the **a** $M_{{{J} /\psi } \mu }$ and **b** $k$-factor model due to the contribution of feed-down modes after the selection. The contribution to the $M_{{{J} /\psi } \mu }$ distribution from ${B} _{c} ^+ \rightarrow {{J} /\psi } \mu ^+ \upnu _\mu$ decays alone is shown by the *black solid curve*, while the inclusion of each modelled feed-down contribution is shown by the *shaded* areas according to the legend. The mean and r.m.s. width of the $k$-factor distribution are shown as a function of $M_{{{J} /\psi } \mu }$ before (*solid line*) and after (*dashed line*) the inclusion of feed-down modes

The signal model must also consider feed-down modes. Their contribution is expected to bias the measured lifetime by modifying the $M_{{{J} /\psi } \mu }$ distribution towards lower values and, to a lesser extent, by affecting the $k$-factor distribution. The modes explicitly included in the model are semileptonic ${B} _{c} ^+$ decays to the higher charmonia states $\psi (2S)$, $\chi _{cJ} ~(J=0,1,2)$ and $h_{c}$, subsequently decaying to a ${{J} /\psi } X$ final state, and the $B_c^+ \rightarrow {{J} /\psi } \uptau ^+ \upnu _\tau$ decay followed by $\uptau ^+ \rightarrow \mu ^+ \upnu _\mu \overline{\upnu } _\tau$. Theoretical calculations give the decay widths of these decay chains relative to ${B} _{c} ^+ \rightarrow {{J} /\psi } \mu ^+ \upnu _\mu$ to be 3.0% for the $\psi (2S)$ mode [35], 3.3% for the sum of $\upchi _{c}$ and $h_c$ contributions [38–41], and 4.4% for ${{J} /\psi } \uptau ^+ \upnu _\tau$ decays [1]. The first two of these are subject to large uncertainties, which are considered in the systematic uncertainty. The contribution of the feed-down modes after the selection is found to be small, as shown in Fig. 2.

Other possible feed-down contributions are the abundant ${B} _{c} ^+ \rightarrow {B} ^0_{s} \mu ^+ \upnu _\mu$ decay mode, followed by the ${B} ^0_{s} \rightarrow {{J} /\psi } X$ decay, and decays to ${{J} /\psi } {D} ^+ _{(s)}$ final states followed by the semileptonic decay of the charmed meson. These channels are studied using simulated events and found to be negligible, mainly because of the softer $p_{\mathrm{T}}$ spectrum of the bachelor muon, and the long-lived intermediate particles causing the reconstructed three-muon vertex to be of poor quality.

## Background model

The main background to decays of long lived particles to three muons is expected to be due to hadrons misidentified as muons and combined with a ${{J} /\psi }$ meson from the same vertex, hereafter referred to as *misidentification background*. Other sources of background, with a correctly identified bachelor muon, are either due to false ${{J} /\psi }$ candidates (*fake* ${{J} /\psi }$ *background* in the following), or associations of a genuine ${{J} /\psi }$ meson and a real bachelor muon not originating from ${B} _{c} ^+$ decays. In the latter case, the two particles can both be produced at the PV (*prompt background*), produced at different vertices and randomly associated (*combinatorial background*), or produced at the same detached vertex ($B\rightarrow 3\mu$ *background*). The yield and PDF of each contribution is modelled from data, with the exception of the last two categories, where simulation is used.

The misidentification background can be accurately predicted from data as no identification requirements are imposed on the bachelor muon by the trigger. By also removing such requirements from the offline selection, a ${{J} /\psi }$-*track* sample consisting of $5.5\times 10^6$ candidates, dominated by ${{J} /\psi }$-hadron combinations, is obtained. The misidentification background is modelled by weighting each candidate in this sample by $W$, the probability to misidentify a hadron as a bachelor muon candidate. This is defined as the average over hadron species $h$ of the misidentification probability $W_h$ for the given species, each being weighted by the probability $P_h$ for the track to be a hadron $h$

5 $$\begin{aligned} W = \sum _{h = K, \pi , p} P_h(\eta , p_{h}, I) W_h(\eta , p_{h}, N_t), \end{aligned}$$

where $$h$$ can be a kaon, a pion or a proton. The quantities $$P_h$$ and $$W_h$$ are measured using calibration samples, as functions of the most relevant variables on which they depend. For $$P_h$$, these are the track momentum $$p_{h}$$, its pseudorapidity $$\eta $$, and the impact parameter $$I$$ with respect to the PV. The dependence on $$I$$ arises because particles produced at the collision vertex will prevail around the PV position, while $${b} $$-hadron decays dominate the events with a sizeable $$I$$ value.

For $W_h$, the variables are $p_{h}$, $\eta$ and the number of tracks in the event $N_t$, since the particle identification performance, notably for the Cherenkov detectors, is affected by the density of hits. The contribution from cases where the bachelor track in the ${{J} /\psi }$-track sample is a lepton is neglected, since its effect on the predicted background yield is small compared to the final statistical and systematic uncertainties. Calibration samples consist of selected ${D} ^{*+} \rightarrow \pi ^+ {D} ^0 ({K} ^- \pi ^+)$ decays for kaons and pions, and $\Lambda \rightarrow {p} \pi ^-$ decays for protons. The residual background to these selections, at the level of a few per cent, is subtracted using events in the sidebands of the $D$ or $\Lambda$ mass distributions.

**Fig. 3:**
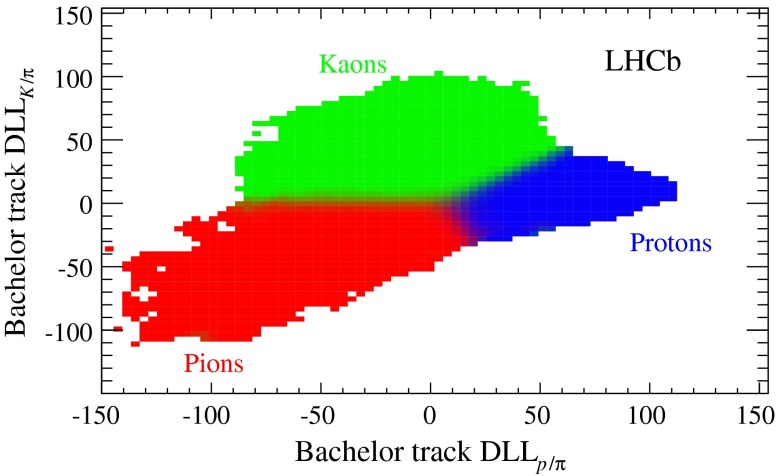
Result of a fit in the ($\mathrm {DLL} _{p/\pi }, \mathrm {DLL} _{K/\pi }$) plane to determine the fractions of hadron species in the ${{J} /\psi }$-track sample. The *colour* of each bin is built as a combination of *red*, *green* and *blue* proportional to the fitted fractions of pions, kaons and protons, respectively

**Fig. 4:**
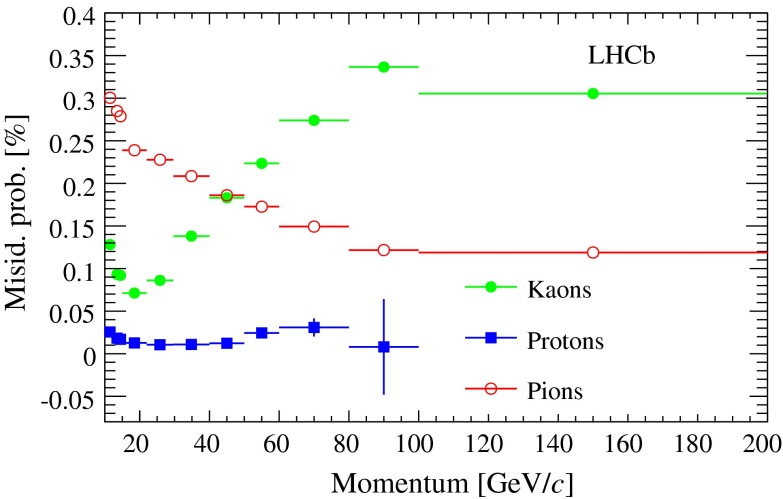
Probability for pions, kaons and protons to be misidentified as muons, as a function of the particle momentum

The hadron fractions are determined in each bin from fits to the two-dimensional distribution of the particle identification variables $$\mathrm {DLL} _{K/\pi }$$ and $$\mathrm {DLL} _{p/\pi }$$ in the $${{J} /\psi }$$-track sample. The discriminating power achievable with these variables is illustrated in Fig. 3. The misidentification probabilities $$W_h$$ are obtained by applying the muon identification criteria to the calibration samples. The result as a function of momentum, averaged over $$\eta $$ and $$N_t$$, is shown in Fig. 4. The approximately exponential dependence for pions is due to decays in flight, while the Cherenkov detectors provide a better identification performance for low momentum kaons. The average value of $$W$$ is found to be 0.20%, corresponding to an expected yield of $$10\,978 \pm 110$$ misidentified candidates, where the uncertainty is statistical only. The two-dimensional ($$t_{\mathrm {ps}}$$, $$M_{{{J} /\psi } \mu }$$) PDF is obtained from the $${{J} /\psi }$$-track events weighted according to Eq. 5. A yield of $$1686 \pm 90$$ misidentified candidates is predicted in the detached region (defined as $$t_{\mathrm {ps}} >150$$ fs), due to $${b} $$-hadron decays. The model is validated by comparing it with the prediction from a simulated sample of events containing a $$B \rightarrow {{J} /\psi } X$$ decay, where $$B={B} ^+ ,{B} ^0,{B} ^0_{s} $$. The yield and PDF shape are primarily due to a set of exclusive $$B$$ meson decays, the most important ones being $${B} ^0 \rightarrow {{J} /\psi } {K} ^{*0} $$ and $${B} ^+ \rightarrow {{J} /\psi } {K} ^+ $$.

**Fig. 5:**
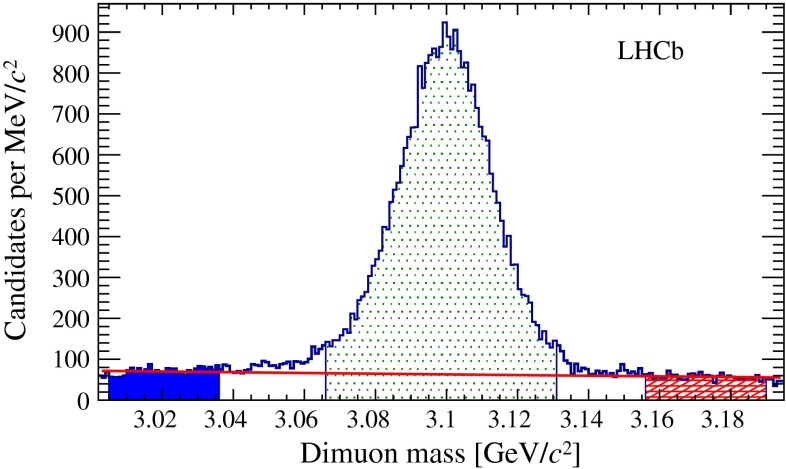
Dimuon mass distribution for the ${{J} /\psi }$ candidates. The selected signal region is shown by the central *light-shaded* area. The sidebands used for the estimation of the fake ${{J} /\psi }$ background are shown by the *dark-shaded* areas, and the function modelling such background by the *solid red* curve

The fake ${{J} /\psi }$ background is modelled using the ${{J} /\psi }$ mass sidebands, as illustrated in Fig. 5. The expected yield is obtained by extrapolating the distribution from the sidebands assuming an exponential behaviour.

The ($t_{\mathrm {ps}}$, $M_{{{J} /\psi } \mu }$) distribution is found to be statistically consistent in the two sidebands. Since the two variables are found to be correlated, a two-dimensional model is used. To reduce the fluctuations due to the limited sample size, a smoothing based on kernel estimation [42] is applied to the observed two-dimensional distribution. The candidates with a fake ${{J} /\psi }$ and a misidentified bachelor muon are already taken into account in the misidentification background category. Their yield and PDF shape are estimated with the same technique used for the misidentification background, namely by weighting ${{J} /\psi }$-track events in the sideband regions according to Eq. 5, and are subtracted from the fake ${{J} /\psi }$ model. After this correction, the fake ${{J} /\psi }$ background yield is predicted to be 2994 $\pm$ 109 candidates.

The prompt background component is important for decays close to the PV, while it is suppressed in the detached region, where most of the signal is expected. To constrain the effects of the tails of the $t_{\mathrm {ps}}$ distribution for prompt background events, the PV region is included in the fit, allowing the yield and shape parameters of the prompt background to be determined from data. Alternative fits with a detachment requirement are used as checks for systematic effects. The $t_{\mathrm {ps}}$ distribution is modelled by a Gaussian function, whose parameter values are left free to vary in the fit. The $M_{{{J} /\psi } \mu }$ distribution is obtained from the events in the prompt region, requiring $-500 < t_{\mathrm {ps}} < 10$ fs to remove the signal component, making the identification requirements for the bachelor muon more stringent to suppress the contamination from the misidentification background. Since no correlations are found between $t_{\mathrm {ps}}$ and $M_{{{J} /\psi } \mu }$ in simulated events, the two-dimensional model is obtained by multiplying the PDFs of the two variables.

The combinatorial background is modelled using a sample of $$18$$ million events containing a $$B \rightarrow {{J} /\psi } X$$ decay, simulated according to the known $${b} $$-quark fragmentation fractions [43] and the $$B$$ meson branching fractions to these states, and additional simulated samples of $$\Lambda ^0_{b} \rightarrow {{J} /\psi } \Lambda $$ and $$\Lambda ^0_{b} \rightarrow {{J} /\psi } {p} {K} ^- $$ decays to estimate the contribution from $${b} $$ baryons. The measured value of the $$\Lambda ^0_{b} $$ fragmentation fraction [44] is used, and the inclusive $$\Lambda ^0_{b} \rightarrow {{J} /\psi } X$$ branching fraction is assumed to equal that in $$B$$ meson decays. The modest sample surviving the selection is used to model the $$t_{\mathrm {ps}}$$ and $$M_{{{J} /\psi } \mu }$$ distributions, neglecting their correlation. The $$t_{\mathrm {ps}}$$ distribution is parametrised with the sum of two exponential functions, while the mass distribution is modelled using the kernel estimation technique. The number of events obtained from simulation is scaled according to the measured $${{J} /\psi }$$ production cross-section from $${b} $$ decays [45], the number of simulated events and the integrated luminosity of the data sample. The resulting yield is $$974 \pm 168$$ candidates, where the uncertainty is statistical. Sizeable systematic uncertainties are assigned to this simulation-based estimation, as discussed in Sect. 7.

Simulated samples are also used to evaluate possible irreducible backgrounds from $${b} $$ hadrons (different from $${B} _{c} ^+$$) decaying to $${{J} /\psi } \mu ^+ X$$ final states where all three muons are produced at the same vertex. The only decay mode with a non-negligible contribution is found to be $${B} ^0_{s} \rightarrow {{J} /\psi } (\mu ^+\mu ^-)\phi (\mu ^+\mu ^-)$$, from which fewer than 20 events are expected. This $$B\rightarrow 3\mu $$ background represents only 2% of the combinatorial background and is merged into that category in the following.

Finally, the background model includes a component to describe events having an incorrectly associated PV, resulting in a faulty reconstruction of the pseudo-proper time. These events are modelled by associating the candidates with the primary vertices measured in the previous selected event. The PDF is obtained from two-dimensional kernel estimation smoothing, while the yield is left free in the fit.

## Fit and results

**Fig. 6:**
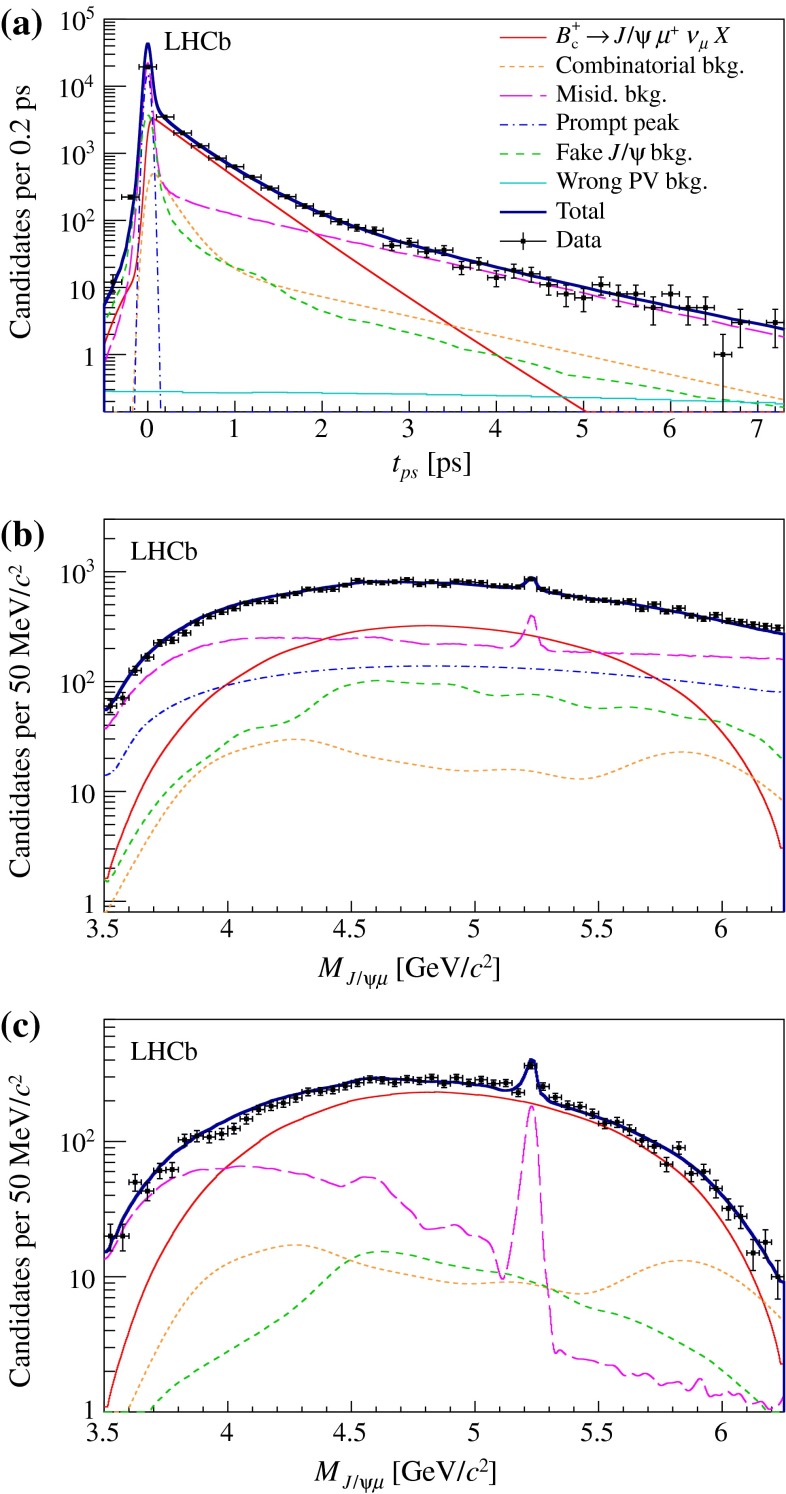
Result of the two-dimensional fit of the ${B} _{c} ^+ \rightarrow {{J} /\psi } \mu ^+ \upnu _\mu X$ model. Projections of the total fit function and its components are shown for **a** the pseudo-proper time, **b** the mass of all events, and **c** the mass of the detached events ($t_{\mathrm {ps}} > 150$ fs)

The ${B} _{c} ^+$ lifetime $\tau$ is determined from a maximum likelihood unbinned fit to the ($t_{\mathrm {ps}}$, $M_{{{J} /\psi } \mu }$) distribution of the selected sample, in the range $-1.5 <t_{\mathrm {ps}} <8$ ps and $3.5 < M_{{{J} /\psi } \mu } < 6.25$ ${\mathrm {\,GeV\!/}c^2}$. To avoid inadvertent experimenter bias, an unknown offset is added to the result for $\tau$, and is removed only after the finalization of the event selection and analysis procedure. The other free parameters of the fit are the mean and width of the $t_{\mathrm {ps}}$ resolution function for the prompt background, and the yields for the signal, the prompt background, and the candidates with an incorrectly associated PV. The yield parameters for the other background components are Gaussian-constrained to their predicted values. The total yield is constrained to the number of events in the sample. Figure 6 shows the projected distributions of the two variables, together with the signal and background contributions obtained from the fit.

The fitted number of signal candidates is $8995 \pm 103$. The ${B} _{c} ^+$ lifetime is determined to be $\tau = 508.7\pm 7.7$ fs, where the uncertainty is statistical only. The total number of background candidates is $20\,760 \pm 120$, of which 2585 have $t_{\mathrm {ps}} >150$ fs. In the detached region, signal decays dominate the sample, particularly for $M_{{{J} /\psi } \mu }$ values above 4.5 ${\mathrm {\,GeV\!/}c^2}$. The number of candidates with an incorrectly associated PV is found to be $12 \pm 5$, corresponding to a probability of incorrect association smaller than 0.1%. The fitted mean and width of the prompt peak are $-2.1 \pm 0.9$ and $32.8 \pm$ 0.7 fs, respectively, in excellent agreement with the values obtained from simulation. The correlations between $\tau$ and the other free parameters are all below 20%. Residuals from the fit are consistent with zero in the explored region of the ($t_{\mathrm {ps}}$, $M_{{{J} /\psi } \mu }$) plane.

A goodness-of-fit test is performed by dividing the region into 100$\times$100 equally sized bins and computing a $\chi ^2$ from the bins for which the expected event yield is larger than 0.5. The resulting $p$-value is 0.20. The method is validated using a set of pseudo-experiments generated according to the fitted model, where the $p$-value distribution is found to be consistent with the expected uniform distribution in [0, 1]. Tests on pseudo-experiments also show that the fit provides unbiased estimates for the lifetime and its statistical uncertainty.

## Systematic uncertainties and checks

The assigned systematic uncertainties to the ${B} _{c} ^+$ lifetime determination, described in the following, are summarised in Table 1. Since limited experimental information is available on semileptonic ${B} _{c} ^+$ decays, uncertainties on the assumed signal PDF are estimated by constraining generic model variations using the distributions observed in data, rather than relying on theoretical predictions. The ${B} _{c} ^+$ production spectra obtained with the Bcvegpy generator are validated using the measured spectra in ${B} _{c} ^+ \rightarrow {{J} /\psi } \pi ^+$ decays and found to be in good agreement. Linear deformations are applied to the rapidity and momentum spectra by reweighting the simulated events. The fit is repeated after applying the maximum deformations indicated by the comparison with the data distributions. The effect on the lifetime is found to be within $\pm$1.0 fs.

**Table 1:** Systematic uncertainties on the ${B} _{c} ^+$ lifetime

Source	Assigned systematic (fs)
${B} _{c} ^+$ production model	1.0
${B} _{c} ^+$ decay model	5.0
Signal resolution model	1.3
Prompt background model	6.4
Fake ${{J} /\psi }$ background yield	0.4
Fake ${{J} /\psi }$ background shape	2.3
Combinatorial background yield	3.4
Combinatorial background shape	7.3
Misidentification background yield	0.8
Misidentification background shape	1.2
Length scale calibration	1.3
Momentum scale calibration	0.2
Efficiency function	2.6
Incorrect association to PV	1.8
Multiple candidates	1.0
Fit validation	0.5
Quadratic sum	12.4

**Fig. 7:**
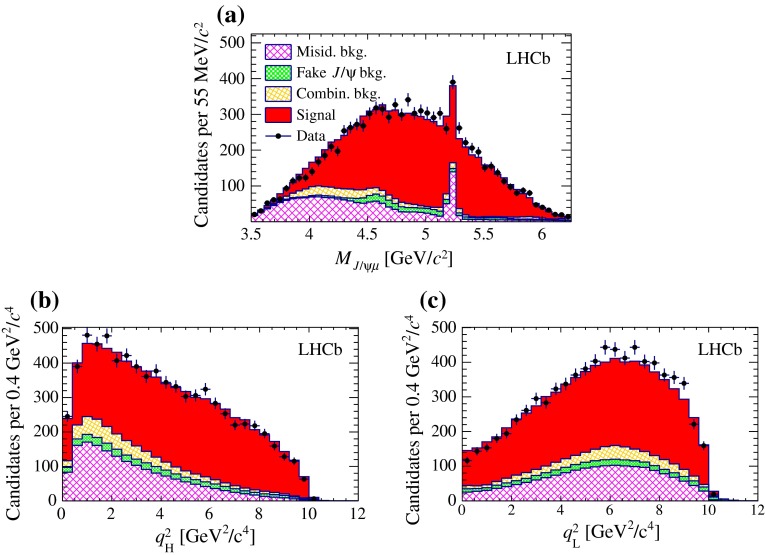
Binned distributions of **a** $M_{{{J} /\psi } \mu }$, and **b**, **c** the two $q^2$ solutions for events in the detached region. The modelled contributions for misidentification background (*hatched dark violet*), fake ${{J} /\psi }$ background (*filled light green*), combinatorial background (*hatched light orange*) and signal (*filled dark red*), are shown, stacked on each other. *Markers* representing data are superimposed. The background yields and PDFs are obtained with the techniques described in Sect. 5, and only the signal yield is obtained from the fit to the data

**Fig. 8:**
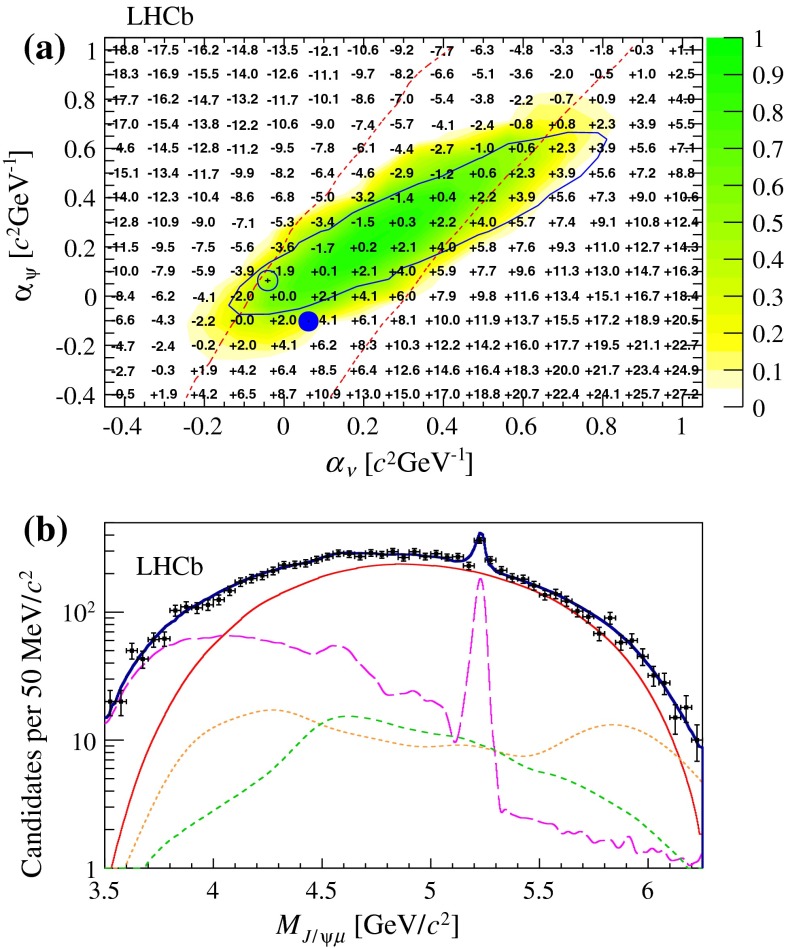
Effect of a generic deformation of the signal model: **a** offset to the lifetime value (expressed in fs) as a function of the deformation parameters; **b** fit projection for the $M_{{{J} /\psi } \mu }$ variable in the detached region after applying the deformation maximising the agreement with data ($\alpha _\psi = \alpha _\nu$ = 0.3 $c^2~\mathrm {\,GeV} ^{-1}$). The colour scale on the *upper* plot indicates the $p$-value of the goodness-of-fit test on the three decay kinematic distributions. The *solid blue* (*dashed red*) lines shows the region having $p$-value greater than 32% for the $M_{{{J} /\psi } \mu }$ ($q^2$) test only. The filled (empty) *blue marker* indicates the deformation parameters that fit best the Ebert (ISGW2) model. The fit components shown on the lower plot follow the legend of Fig. 6

The same technique is used for the uncertainties on the ${B} _{c} ^+ \rightarrow {{J} /\psi } \mu ^+ \upnu _\mu X$ decay model. A generic model of the distribution in the ${{J} /\psi } \mu \nu$ phase space is defined by applying the following transformation to the nominal model $D(M_{{{J} /\psi } \mu } ^2,M_{\mu \nu } ^2)$

6 $$\begin{aligned}&D'(M_{{{J} /\psi } \mu } ^2,M_{\mu \nu } ^2) = D(M_{{{J} /\psi } \mu } ^2,M_{\mu \nu } ^2)\nonumber \\&\quad \times \exp (\alpha _\psi M_{{{J} /\psi } \mu } + \alpha _\nu M_{\mu \nu }), \end{aligned}$$

where $M_{\mu \nu } ^2=q^2$ is the squared mass of the $\mu \nu$ combination. The deformation parameters $\alpha _\psi$ and $\alpha _\nu$ represent generic imperfections of the model for the decay form factors and feed-down contributions. The exponential deformation is chosen to have positive weights while keeping an approximately linear deformation in the masses for small values of the deformation parameters. Partial reconstruction of the decay, using the measured flight direction of the ${B} _{c} ^+$ meson and its known mass value, is used to determine its momentum up to a two-fold ambiguity. The agreement between the deformed model and the data is evaluated, using the signal-enriched detached sample, from the distributions of $M_{{{J} /\psi } \mu }$ and of the two $q^2$ solutions $q^2_{\mathrm H}$ ($q^2_{\mathrm L}$) obtained using the higher (lower) solution for the ${B} _{c} ^+$ momentum. The comparison for the nominal model is shown in Fig. 7. Figure 8a shows the results of goodness of fit tests obtained when varying the deformation parameters. The agreement is assessed by performing a $\chi ^2$ test on each of the three distributions.

Among the models compatible with data at 90% confidence level (combined $p$-value $>$ 0.1), variations of the ${B} _{c} ^+$ lifetime are within $\pm$5.0 fs, which is assigned as systematic uncertainty. It can be noted, by comparing Fig. 8b with Fig. 6c, that the fit quality in the mass projection is significantly improved after applying the deformation that maximises the combined $p$-value.

As a consistency check, the model is also varied within the uncertainties evaluated by comparing available theoretical predictions for the $${B} _{c} ^+ \rightarrow {{J} /\psi } \mu ^+ \upnu _\mu $$ form factors and feed-down contributions. When the signal model is built using the alternative samples of simulated $${B} _{c} ^+ \rightarrow {{J} /\psi } \mu ^+ \upnu _\mu $$ decays generated with the Ebert and ISGW2 form-factor models, the lifetime changes by $$+2.0$$ fs and $$ -1.5$$ fs, respectively, consistent with the model-independent evaluation. Indeed, the deformation parameters corresponding to the best approximation of the alternative models, shown in Fig. 8a, are compatible with data with a confidence level in excess of 90%. For the feed-down contributions, the relative decay widths with respect to the $${B} _{c} ^+ \rightarrow {{J} /\psi } \mu ^+ \upnu _\mu $$ decay are varied according to the range of values predicted in Refs. [7, 35, 36, 38, 39, 46–49]. More conservatively, each modelled component is varied in turn by $$\pm $$100% in order to take into account possible smaller contributions, such as non-resonant $$B_c^+ \rightarrow J/\psi \mu \nu \pi ^0$$ decays, which have not been modelled explicitly and whose PDF shapes are intermediate between the considered ones. The maximum variation with respect to the nominal fit is 0.3 fs.

Several effects concerning the reconstruction of signal events are considered. The resolution model for the signal is varied using a quadruple Gaussian instead of the nominal triple Gaussian model. The lifetime variation is $$ -1.3$$ fs, which is assigned as the systematic uncertainty from this source. The number of $$M_{{{J} /\psi } \mu }$$ bins used for the $$k$$-factor parametrization is varied to evaluate the effect of the discretization. Results obtained with more than ten bins are stable within $$\pm 0.1$$ fs and the effect is neglected.

**Fig. 9:**
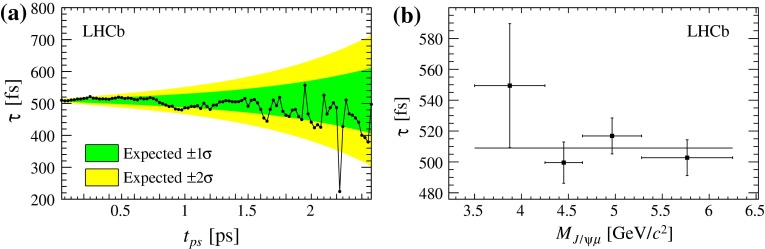
Lifetime results obtained after reducing the range of the $t_{\mathrm {ps}}$ and $M_{{{J} /\psi } \mu }$ variables **a** removing the events with $t_{\mathrm {ps}}$ lower than the threshold reported on the $x$-axis and, **b** dividing events in bins of $M_{{{J} /\psi } \mu }$. The dark (*light*) *shaded* band on **a** shows the expected $\pm$1(2) statistical standard deviation ($\sigma$) of the lifetime variation due to the reduced sample size. The *horizontal line* on **b** shows the lifetime result of the nominal fit

A possible systematic bias related to the prompt background model is explored by performing fits with a minimum requirement on the $t_{\mathrm {ps}}$ value. The results, shown in Fig. 9a, are consistent with the expected fluctuations due to the reduced size of the sample. The lifetime variation obtained with the $t_{\mathrm {ps}} >150~$fs requirement, which removes most of the prompt candidates, is $+6.4$ fs, corresponding to 1.5 times the expected statistical error, and is conservatively taken as the systematic uncertainty. The fitted lifetime value changes within this uncertainty when modifying the $t_{\mathrm {ps}}$ resolution function, using a triple Gaussian shape obtained from simulation instead of the single Gaussian with free parameters used in the nominal fit, or when using the $M_{{{J} /\psi } \mu }$ distribution predicted using simulated events with prompt ${{J} /\psi }$ production.

For the fake ${{J} /\psi }$ background, the expectation value of its yield is varied within its systematic uncertainty. The uncertainty on the PDF shape is studied using the two alternative models obtained using only one of the two ${{J} /\psi }$ mass sidebands. The observed offsets are within $\pm 2.3$ fs.

Since the combinatorial contribution is the only background source whose model relies on simulation, data-driven checks are performed to evaluate the uncertainty on its predicted yield. The yield of detached candidates before the bachelor muon identification requirements, which is expected to be dominated by $${b} $$-hadron decays, is measured and found to differ by 35% from the value predicted by the simulation. To account for a further uncertainty related to the efficiency of the muon identification criteria, a systematic uncertainty of $$\pm 50\%$$ is assigned on the combinatorial background yield. Another check is performed by comparing the event yields in data and simulation for candidates with $$M_{{{J} /\psi } \mu }$$ values above the $${B} _{c} ^+$$ mass, where only combinatorial background is expected. For this check, an additional requirement $$p_T(J/\psi ) > 3$$ $${\mathrm {GeV\!/}c}$$ is applied on simulated data, since data are filtered with such a requirement in this mass region. The observed event yield is $$221 \pm 14$$ events, and the predicted yield is $$201 \pm 73$$. The pseudo-proper time and mass distributions are also found to agree. The quoted uncertainty on the yield corresponds to $$\pm $$3.4 fs on $$\tau $$. The uncertainty on the PDF is dominated by the shape of the $$t_{\mathrm {ps}}$$ distribution. A single exponential rather than a double exponential function is used, and the parameters of the nominal function are varied within their statistical uncertainty. The maximum variation is $$ -7.3$$ fs.

For the misidentification background, an alternative fit is performed allowing its yield to vary freely, instead of being Gaussian constrained to its predicted value. The exercise is repeated using only detached events. The resulting yields are found to be compatible with the expected ones, and the maximum $$\tau $$ variation of $$+0.8$$ fs is taken as systematic uncertainty. The accuracy of the PDF model is limited by the size of the calibration and $${{J} /\psi }$$-track samples, since the misidentification probability $$W$$ of Eq. 5 is parametrised in bins of several variables. The effect of the uncertainty in each bin is estimated by simulating 1000 alternative PDFs after applying random offsets to the $$W$$ values, according to their statistical uncertainty. The maximum variation of the lifetime is $$ -1.2$$ fs.

Systematic biases on the reconstruction of the pseudo-proper time scale can be produced by miscalibration of the detector length or momentum scale, and by a dependence on the decay time of the reconstruction and selection efficiency $$\epsilon $$. All these effects have been evaluated using simulation and control samples in previous studies. The uncertainty on position effects is known [50] to be dominated by the calibration of the longitudinal scale. The resulting effect on $$\tau $$ is within $$\pm $$1.3 fs. The momentum scale is varied within its uncertainty [51] and the effect is found to be negligible. If the dependence of the efficiency $$\varepsilon $$ on the decay time is linearly approximated as $$\varepsilon (t) \propto (1+\beta t)$$, the bias on the lifetime is about $$\beta \tau ^2$$. According to the simulation used in this study, the value of $$\beta $$ is compatible with zero within a statistical uncertainty of 6 ns$$^{-1}$$. An uncertainty of $$\pm 10 $$ ns$$^{-1}$$ on $$\beta $$ is conservatively assigned, based on data-driven studies of the effects contributing to $$\beta $$ for some exclusive $${b} $$-hadron to $${{J} /\psi } X$$ modes [52]. The corresponding systematic uncertainty on $$\tau $$ is $$\pm 2.6$$ fs.

To estimate the uncertainty on the modelling of events with an incorrectly associated PV, the fit is repeated removing events where more than one PV are compatible with the candidate decay. The lifetime changes by $+1.8$ fs. A possible effect due to multiple candidates in the same event is studied by introducing an explicit bias, retaining only the candidate with the lowest or highest $t_{\mathrm {ps}}$ value. The bias is found to be within 1.0 fs. Finally, the fit procedure is validated using 300 simulated pseudo-experiments generated according to the nominal fit model. The average value of the fitted lifetime agrees with the generated value within the statistical uncertainty of $0.5$ fs.

The sum in quadrature of the mentioned contributions is 12.4 fs. Several further consistency checks have been performed to probe residual biases not accounted for by the assigned systematic uncertainty. To check the reliability of the prediction for the $k$-factor distribution, including the reconstruction effects, a sample of ${B} ^0 \rightarrow {{J} /\psi } {K} ^+ \pi ^-$ decays is reconstructed with or without the pion in the final state. Using the information from the fully reconstructed decay, the distribution of the $k$-factor, defined in this case as the ratio of the two reconstructed quantities $t_{\mathrm {ps}}$/$t$, is measured from data and compared to the prediction from simulation. After reweighting for the observed distribution of the ${{J} /\psi } {K} ^+$ mass, the distributions are found to agree well, and the average $k$-factor is predicted to better than 0.1%, corresponding to a bias on the lifetime below $0.5$ fs.

In the selected sample, the high-$$t_{\mathrm {ps}}$$ tail of the distribution is dominated by $${b} $$-hadron decays. To check for a possible mismodelling of this background, the analysis is repeated varying the maximum $$t_{\mathrm {ps}}$$ requirement between 2 and 8 ps. The resulting lifetime variations are within $$\pm $$1.5 fs, which is compatible with the expected statistical fluctuations. The fit is also performed in four bins of $$M_{{{J} /\psi } \mu }$$, since the background and feed-down contributions vary strongly with mass and become very small above the $${B} ^+ $$ mass. As shown in Fig. 9b, no significant differences are found among the four results. Another check performed is to relax the requirement on vertex quality from the nominal $$\chi ^2 <3$$ up to $$\chi ^2 <9$$. For this test, the yield of combinatorial background is allowed to vary. It is found to be compatible with the expected yield, and to be proportional to the vertex $$\chi ^2$$ threshold, as predicted by the simulation. The corresponding changes in $$\tau $$ are within $$\pm $$2.5 fs and are also compatible with the expected statistical fluctuations. Finally, the analysis is repeated after splitting the sample into two parts, according to the polarity of the spectrometer magnet, which is inverted at regular intervals during the data taking period. The difference between the $$\tau $$ results from the two polarities is consistent with zero within one standard deviation.

## Conclusions

Using ${B} _{c} ^+ \rightarrow {{J} /\psi } \mu ^+ \upnu _\mu X$ semileptonic decays, reconstructed with the LHCb detector from $pp$ collision data corresponding to an integrated luminosity of $2 \text{ fb }^{-1}$, the lifetime of the ${B} _{c} ^+$ meson is measured to be

$$\begin{aligned} \tau = 509\pm 8\mathrm {\,(stat)} \pm 12(\hbox {syst})\, \text { fs}. \end{aligned}$$

This is the most precise measurement of the $B_c^+$ lifetime to date. It is consistent with the current world average [10] and has less than half the uncertainty. This result will improve the accuracy of most ${B} _{c} ^+$ related measurements, and provides a means of testing theoretical models describing the ${B} _{c} ^+$ meson dynamics. Further improvements are expected from the LHCb experiment using ${B} _{c} ^+ \rightarrow {{J} /\psi } \pi ^+$ decays, where systematic uncertainties are expected to be largely uncorrelated with those affecting the present determination.

## Footnotes

1 The inclusion of charge conjugate states is always implied throughout this paper.
